# Research on the Electrical Tree Deterioration Characteristics of Silicone Gel and Silicone Rubber Under Pulsed Electric Field

**DOI:** 10.3390/gels11040253

**Published:** 2025-03-28

**Authors:** Cong Zhang, Xiangze An, Qingfa Li, Jian Wu, Zhe Xu, Usama Khaled, Dongxin He, Lin Zhu

**Affiliations:** 1 School of Electrical Engineering, Shandong University, Jingshi Road 17923, Jinan 250061, China; 202485009043@sdu.edu.cn (C.Z.); 202000190125@mail.sdu.edu.cn (X.A.); 202334714@mail.sdu.edu.cn (Q.L.); 2 Department of the Xirui Green Electric Limited Company, Suzhou 215021, China; wjawja63@outlook.com; 3 Department of the State Grid Shandong Electric Power Company Extra High Voltage Company, Qingdao 266000, China; epezhangyl@mail.sdu.edu.cn; 4 Department of Electrical Engineering, Faculty of Energy Engineering, Aswan University, Aswan 81528, Egypt; usamakhaled@energy.aswu.edu.eg; 5 School of Materials Science and Engineering, Shandong University, Jingshi Road 17923, Jinan 250061, China

**Keywords:** pulsed electric field, silicone gel, electrical tree

## Abstract

Silicone gel and silicone rubber are widely used in packaging insulation because of their high comprehensive performance. Nevertheless, the special deterioration mechanism under pulsed electric fields is not yet clear and needs to be studied in depth. This study has successfully built an experimental platform of the electrical tree under a thermal coupled pulsed electric field. Moreover, the effects of the pulse edge time, repetition frequency, and temperature on the tree initiation voltage, intuitive development morphology, and fractal dimension of the electrical tree are also investigated, respectively. In conclusion, silicone rubber has a higher insulation strength, while silicone gel has a certain self-recovery performance. The aim of the study is to realize the electrical tree deterioration characteristics of silicone gel and silicone rubber. The increase in repetition frequency, the decrease in edge time, and the increase in temperature all contribute to the initiation and growth of the electrical tree from different degrees and angles, making the electrical tree transform between a fine, dendritic, clumped, and pine-like shape.

## 1. Introduction

The high voltage change rate, high-frequency, and high-temperature harsh operating conditions of high-voltage semiconductor devices require their packaging materials to have a higher insulation performance [1,2,3,4,5]. A silicone elastomer is divided into silicone rubber and silicone gel. Silicone rubber has a high degree of crosslinking, high molecular mass, and strong intermolecular forces, exhibiting excellent electrical insulation and high-temperature resistance [6]. However, due to its relatively high elastic modulus, and poor thermal and mechanical strain capacity, it is easy to create gaps between semiconductor devices under the action of high-temperature and mechanical stress, resulting in a decrease in insulation strength [7]. In contrast, silicone gel has a low elastic modulus, and strong stress strain capacity and adhesion ability with devices, so it can closely fit with semiconductor devices to ensure the packaging insulation performance and buffer protection of devices under stress; but the crosslinking degree of silicone gel is low, the force between molecular chains is weak, and its thermal stability is poor [8,9,10,11,12,13,14,15]. Silicone rubber and silicone gel are, respectively, suitable for power semiconductor devices under different operating conditions, so it is necessary to focus on the insulation properties of the two materials and their differences.

An electrical tree is one of the main ways to damage dielectric insulation and cause breakdown [16,17,18]. Research has shown that compared to DC and AC voltages of the same frequency, pulse voltage promotes the initiation and development of an electrical tree in solid dielectric, and also changes the growth morphology of the electrical tree [19,20,21,22,23]. YING L compared and studied the initial characteristics of a crosslinked polyethylene electrical tree under pulse voltage and direct current voltage, and found that under pulse voltage, the accumulation and distribution of homopolar charges do not reach a steady state due to the fast rise rate in voltage; thus, the weakening effect of homopolar charges on the tip electric field was not significant, making it more prone to generating electrical trees [24]. Wang Peng conducted a comparative study under pulse and sinusoidal voltage, and found that when the voltage amplitude and frequency are the same, the probability of initiation and growth length of an epoxy resin electrical tree under repeated pulse voltage are about three times those under sinusoidal voltage; sinusoidal voltage often triggers single-branch electrical branches, while repeated pulse voltage leads to faster and multi-branch electrical trees [25]. Therefore, it is necessary to investigate the electrical tree deterioration characteristics of silicone elastomers under pulsed electric fields.

Current researchers have independently tested and analyzed the electrical tree characteristics of silicone gel and silicone rubber under pulsed electric fields. For the silicone gel, Mancinelli P and Masaharu F conducted comparative tests under sinusoidal and pulse voltage. The results show that the initiation voltage of electrical trees in silicone gel under a square-wave pulse is much lower than that under sinusoidal voltage of the same frequency. They theoretically speculate that the sharp rising edge of the pulse voltage generates stronger material stress on the medium, which also promotes the sustained growth of electrical trees under the pulse electric field, but have not directly studied the effect of the pulse edge time on electrical trees. Meanwhile, both papers have concluded that the increase in voltage frequency promotes the growth of electrical trees [26,27]. Masahiro Sato placed needle electrodes on a glass substrate, and the electrical tree grew perpendicular to the electric field on the surface of the substrate. Observations revealed that the surface discharge patterns in silicone gel differ based on the pulse polarity: under positive pulses, the discharge tracks appear filamentous, whereas under negative pulses, they take on a spindle-like shape. In addition, with the increase in the square-wave pulse frequency and the decrease in the rise time, the cavity length will also increase. It is proved that these phenomena are related to the variation in the air gap discharge intensity [28]. However, the aforementioned results are obtained under room temperature conditions, and further research is needed to explore the electrical treeing behavior of silicone gel under thermally coupled pulsed electric fields.

For silicone rubber, Du Boxue, Zhang Yunxiao, and others conducted deep research on the electrical tree deterioration characteristics of silicone rubber under multi-field coupling [29,30,31]. Research has found that an increase in pulse frequency leads to an increase in partial discharge, which promotes the growth of electrical trees. Compared with negative polarity pulses, the electrical tree is more likely to grow under positive pulses. At low temperatures, silicone rubber gradually enters the glass state from a high elastic state through a crystalline state, resulting in a decrease in molecular chain activity and a significant impact on the electrical tree characteristics. Based on the DC pulse composite voltage and temperature gradient, the growth characteristics of electrical trees were studied. It was found that as the temperature gradient increased, the length, fractal dimension, and cumulative damage of electrical trees increased; but this study mainly focuses on the operating conditions of cable connectors, with pulse edge times of hundreds of microseconds and frequencies of hundreds of hertz, which are inconsistent with the operating conditions of power electronic devices. Although there are also studies on the use of silicone rubber as a semiconductor device packaging material, the applied voltage conditions in these studies are not pulsed voltages. Therefore, when silicone rubber is used in semiconductor device packaging, it is necessary to study its electrical tree deterioration characteristics under nanosecond-level pulse edge and pulse voltage operating conditions at frequencies of tens of kilohertz, which are semiconductor operating conditions [32,33].

In summary, the current research on the electrical tree deterioration characteristics of silicone elastomer under pulsed electric fields is not comprehensive enough. Specifically, for silicone gel, firstly, there is a lack of research on the impact of the pulse edge time on the electrical tree characteristics in the medium, and secondly, the electrical tree deterioration characteristics under thermal coupled pulsed electric fields have yet to be studied. For silicone rubber, it is necessary to study its electrical tree characteristics under high-voltage semiconductor device operating conditions (nanosecond-level pulse edges, tens of kilohertz frequencies, and high temperatures). Due to the lack of current research, this paper built the electrical tree experiment platform under the electro thermal coupled pulsed electric field to explore the electrical tree deterioration characteristics of silicone gel and silicone rubber. A different pulse voltage repetition frequency, rising and falling edge time, and temperature were set, respectively, to study the initiation and development of a silicone electrical tree under different working conditions. The initiation voltage, development form, and fractal dimension of an electrical tree of silicone gel and silicone rubber were compared and analyzed, and the difference in the deterioration characteristics between silicone gel and silicone rubber was evaluated.

## 2. Results and Discussion

### 2.1. The Effect of Frequency

**Figure 1:**
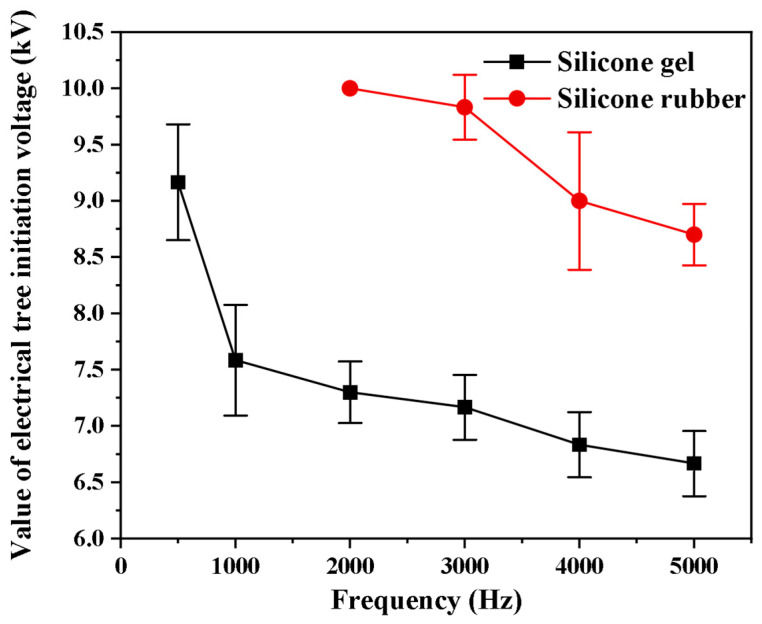
Relation between the mean value of the electrical tree initiation voltage and the pulse voltage repetition frequency.

A negative square-wave pulse voltage with an edge time of 50 ns and duty cycle of 50% was applied to silicone gel and silicone rubber. The frequency was set to 500 Hz, 1000 Hz, 2000 Hz, 3000 Hz, 4000 Hz, and 5000 Hz. At the same time, voltage was applied to five needle–plate electrodes, and three repeatability experiments were carried out for each parameter to observe the tree emergence of 15 needle tips. The tree initiation of silicone gel and silicone rubber under different pulse voltage repetition frequencies is shown (Figure 1). For silicone gel, with the increase in the pulse voltage repetition frequency, the average initiation voltage decreases. For silicone rubber, the treeing voltage also decreases with the increase in the repetition frequency of the pulse voltage, but when the frequency is less than 2 kHz, silicone rubber is not prone to generating an initial electrical tree at voltage levels of 10 kV and below. It can be seen from the comparison that it is easier for the silicone gel to generate the initial electrical tree than the silicone rubber, which means that the withstand voltage and insulation performance of the silicone rubber are higher than those of the silicone gel.

**Figure 2:**
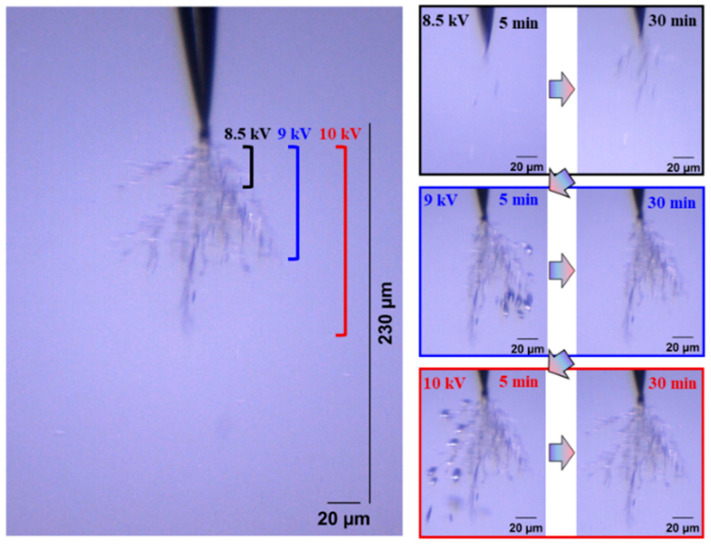
Electrical tree initiation and development process of silicone gel at frequency of 500 Hz.

**Figure 3:**
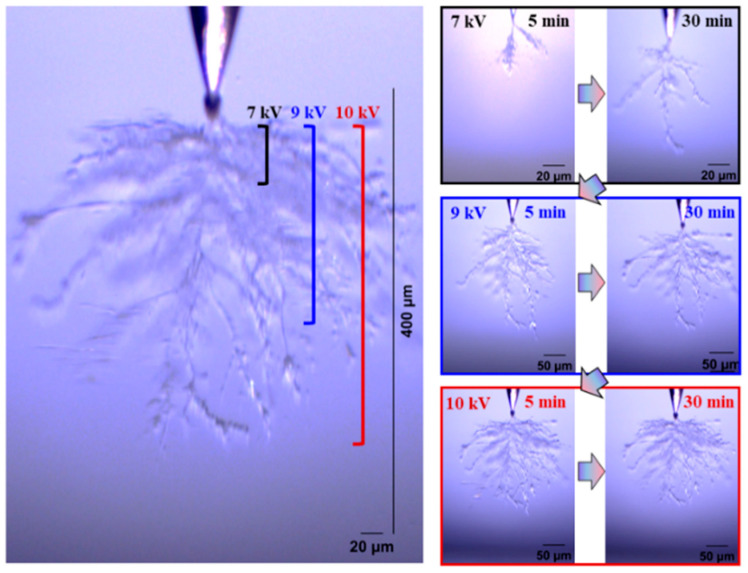
Electrical tree initiation and development process of silicone gel at frequency of 1000 Hz.

The development form of electrical trees is also an index to evaluate the insulation performance of silicone gel and silicone rubber. Electrical trees are divided into fine, dendritic, clumped, and pine-like shape according to their morphology. Firstly, the influence of the frequency on the development of the electrical tree and the difference between the development of the silicone gel and silicone rubber electrical tree were analyzed visually and qualitatively. Figure 2 and Figure 3 show the electrical tree initiation of silicone gel at 500 Hz and 1000 Hz and the development process of silicone gel at 9 kV and 10 kV. With the increase in the voltage level and voltage application time, the electrical tree develops from a fine shape to a dendritic shape, and then to a clumped shape. When the frequency increases from 500 Hz to 1000 Hz, the voltage of the electrical tree of silicone gel decreases, and at the voltage level, the branches of the electrical tree become thicker and develop faster. When the voltage rises to 9 kV, the branches of the electrical tree under high frequency are more numerous and robust, with longer and wider branches than those under low frequency. The electrical tree morphology at low frequencies on the 9 kV voltage level does not vary significantly with the time of voltage application. When the voltage rises to the highest voltage level of 10 kV, the electrical tree under high frequency does not change significantly with the increase in the voltage time; that is to say, at a certain voltage level, the electrical tree of silicone gel will stagnate when the voltage amplitude is constant. When the voltage level is raised again, the electrical tree will further develop. The stepwise voltage application method was employed in this experiment. Direct application of higher voltages may lead to variations in the structure of electrical tree channels, which we plan to explore in future research.

**Figure 4:**
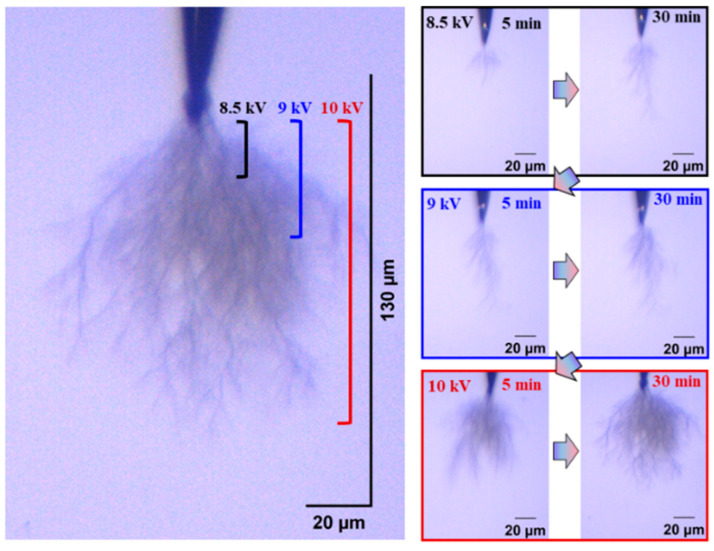
Electrical tree initiation and development process of silicone rubber at frequency of 4000 Hz.

**Figure 5:**
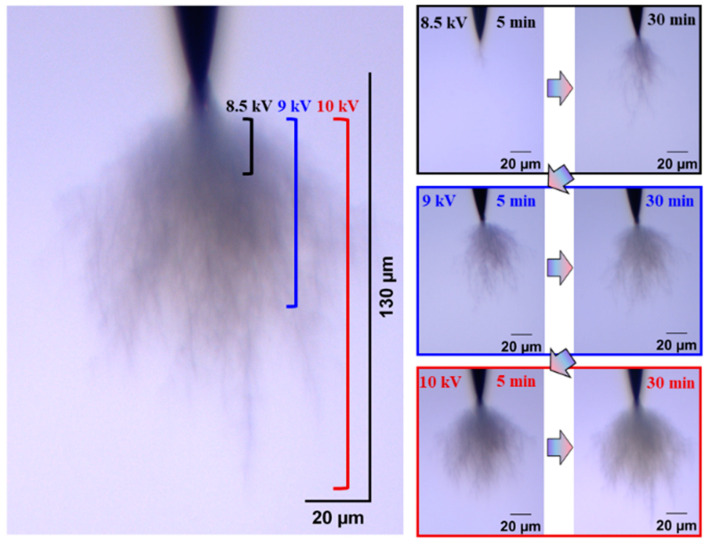
Electrical tree initiation and development process of silicone rubber at frequency of 5000 Hz.

Figure 4 and Figure 5 show the electrical tree initiation and development process of silicone rubber at a frequency of 4000 Hz and 5000 Hz. As can be seen from the figure, the electrical tree of silicone rubber is also a clumped shape. When the frequency is increased from 4000 Hz to 5000 Hz, the development of the electrical tree in silicone rubber with the increasing voltage time is more rapid at the initiation voltage level, and the branches of the electrical tree at high frequencies are denser. When the voltage increases to 9 kV, the electrical tree at low frequencies is relatively sparse, with slow development over time. At high frequencies, the electrical tree is denser than at the initiation voltage levels. Due to the excessive branches and the overlapping of the three-dimensional electrical tree on a two-dimensional screen, the branches of the electrical tree at high frequencies are more blurred. When the voltage increases to 10 kV, the electrical tree branches at low frequencies further develop, and the branches are relatively clear and distinct. However, the morphology of the electrical tree branches at high frequencies becomes more complex and overlapping due to the more intricate branching, resulting in a slightly longer electrical tree branch length at high frequencies compared to low frequencies.

**Figure 6:**
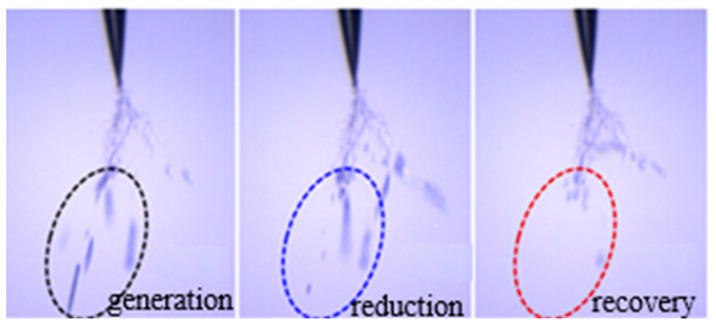
Bubble generation and recovery processes in the electrical tree of silicone gel.

**Figure 7:**
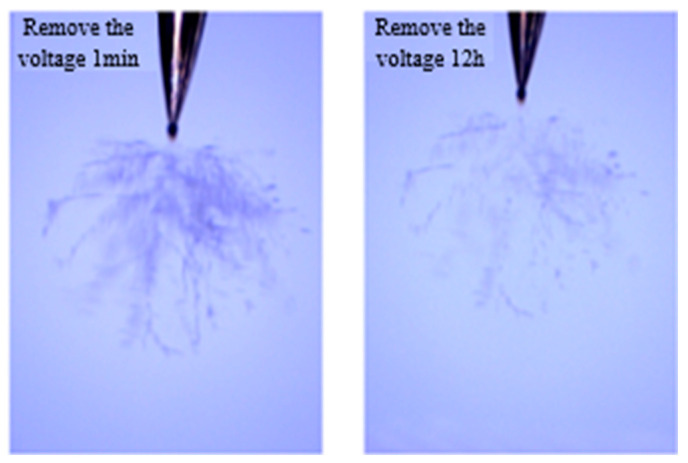
Recovery process of electrical tree channels in silicone gel.

Compared with the development image of silicone gel and silicone rubber, the electrical tree channel of silicone gel is more robust, mainly the air gap channel, which is transparent and reflective under the microscope. However, the electrical tree channels of silicone rubber are thinner and appear black under a microscope. While this may indicate carbonization, it could also be attributed to light scattering or other factors [34]. Silicone gel has a certain self-recovery performance, which is reflected in two aspects. On the one hand, bubbles will appear in the silicone gel during the application of voltage, while some bubbles will disappear in a few seconds, and the original bubble position will not leave the electrical tree channels (Figure 6). On the other hand, after 12 h of placement, the generated electrical tree channel has been restored to some extent (Figure 7). By contrast, silicone rubber does not have a bubble generation and recovery process, as well as an electrical tree channel recovery process. The reason may be that the electrical tree channels in the silicone rubber are carbonized during the generation process, and there are a large number of liquid components in the silicone gel. The discharge in the electrical tree channel causes the liquid component of the silicone gel to expand and produce bubbles, and recovers under the extrusion force of the solid component. The restored electrical tree channel in the silicone gel is an air gap channel, and the unrecovered channel may be a carbonization channel.

When the frequency increases, because the bubbles in the silicone gel will occur at a distance from the needle electrode in a short time, once the grounding plate electrode is directly contacted, an air gap channel will be generated between the two electrodes, leading to the breakdown of the silicone gel. The higher the frequency, the faster the development of the electrical tree, and the greater the probability of breakdown. When the pulse voltage with a repetition rate of 2000 Hz is applied, the silicone gel will break down quickly, so its development process is relatively short. Therefore, only the silicone gel electrical tree growth process from 500 Hz to 2000 Hz is discussed. In contrast, for silicone rubber materials, electrical tree initiation is difficult to achieve even at the maximum output voltage of 10 kV when the frequency is 1000 Hz or below. Therefore, only the growth process of silicone rubber electrical trees from 2000 Hz to 5000 Hz is discussed.

**Figure 8:**
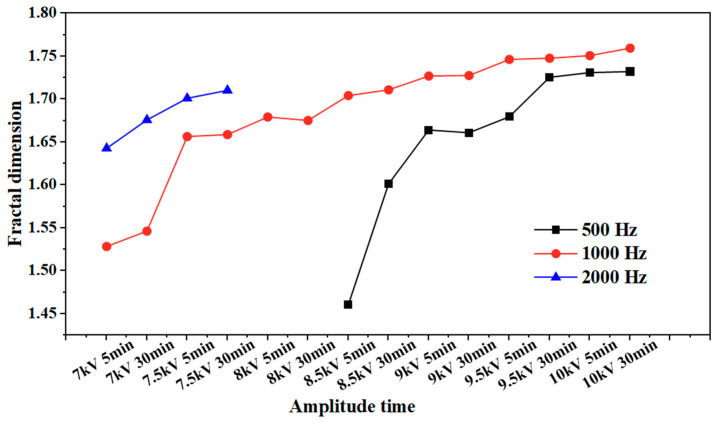
Variation in the electrical tree fractal dimension of silicone gel at different frequencies with voltage amplitude and voltage application time.

**Figure 9:**
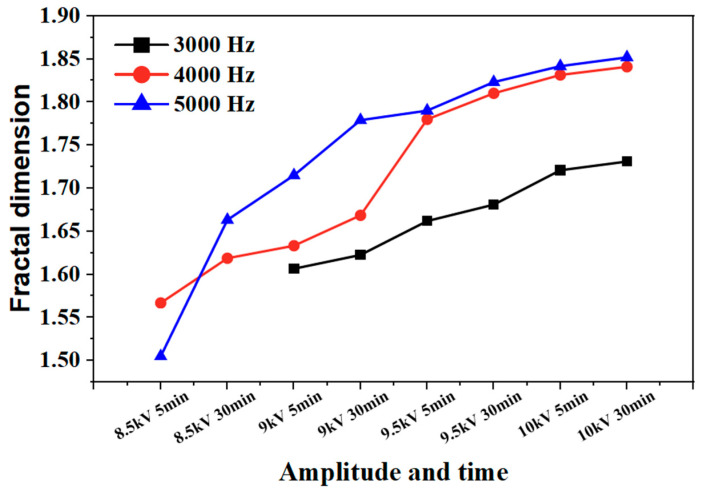
Variation in the electrical tree fractal dimension of silicone rubber at different frequencies with voltage amplitude and voltage application time.

The fractal dimension of the electrical tree development process of silicone gel (Figure 8) and silicone rubber (Figure 9) at different frequencies was calculated. Fractal dimension is a measure of the intensity and complexity of electrical tree development. For silicone gel, the fractal dimension of electrical trees increases gradually with the increase in the voltage amplitude and time. The fractal dimension of electrical trees at a frequency of 500 Hz increases significantly during the period from 8.5 kV for 5 min to 9 kV for 5 min, indicating that electrical trees grow faster at 8.5 kV. The fractal dimension of electrical trees at a frequency of 1000 Hz undergoes a significant mutation during the period from 7 kV for 30 min to 7.5 kV for 5 min, indicating that the electrical tree grows faster at 7.5 kV. At a frequency of 2000 Hz, the electrical tree is prone to breakdown above 8 kV. It can be seen that the fractal dimension of the electrical tree is high at the beginning and rapidly increases, reflecting the rapid growth of electrical trees at high frequencies. After a rapid growth stage, the growth rate slows down. Taking the growth process of electrical trees at 1000 Hz as an example, the fractal increment of electrical trees is very small when the voltage level and time are between 7.5 kV for 5 min and 7.5 kV for 30 min. However, when the voltage level increases from 7.5 kV to 8 kV, the increment is larger, but when the voltage level remains constant at 8 kV, the fractal dimension decreases. The analysis suggests that when the voltage level increases, it will cause the electrical tree to grow faster in a short period of time. If the voltage level remains constant, the electrical tree growth will stop at a certain stage, known as the growth arrest stage, and even recover to a small extent under the influence of material pressure. This phenomenon is related to the recovery of bubbles and air gap channels in Figure 6 and Figure 7. The impact of the pulse voltage repetition frequency on the fractal dimension of electrical trees mainly has two aspects: firstly, if the pulse frequency is high, the fractal dimension of electrical trees increases significantly at lower voltage levels; secondly, the fractal dimension of electrical trees with higher frequencies is always higher than that of electrical trees with lower frequencies. This indicates that the increase in pulse repetition frequency promotes the growth of electrical tree branches.

This effect is also reflected in the change rule of the fractal dimension of silicone rubber. The fractal dimension of electrical trees at a frequency of 5000 Hz increased significantly between 8.5 kV for 30 min and 9 kV for 30 min, while the fractal dimension of electrical trees at a frequency of 4000 Hz increased significantly between 9 kV for 30 min and 9.5 kV for 5 min. Except for the initial stage of electrical trees, the higher the frequency, the greater the fractal dimension of electrical trees, the higher the growth density and complexity throughout the entire stage of electrical tree development.

### 2.2. Effect of Edge Time

A high voltage change rate is an important feature of the operating conditions of high-voltage semiconductor devices. Therefore, it is necessary to change the pulse edge time to change the voltage change rate to explore its influence on the deterioration of silicone gel and silicone rubber electrical trees. Silicone gel has a lower initiation voltage of electrical trees and a more complete development process at 1000 Hz, and the probability of breakdown is also low. Therefore, the pulse repetition frequency of silicone gel should be kept at 1000 Hz for edge time research. Silicone rubber demonstrates a lower electrical tree inception voltage at 5000 Hz. Therefore, the study of silicone rubber is maintained at a repetition frequency of 5000 Hz.

**Figure 10:**
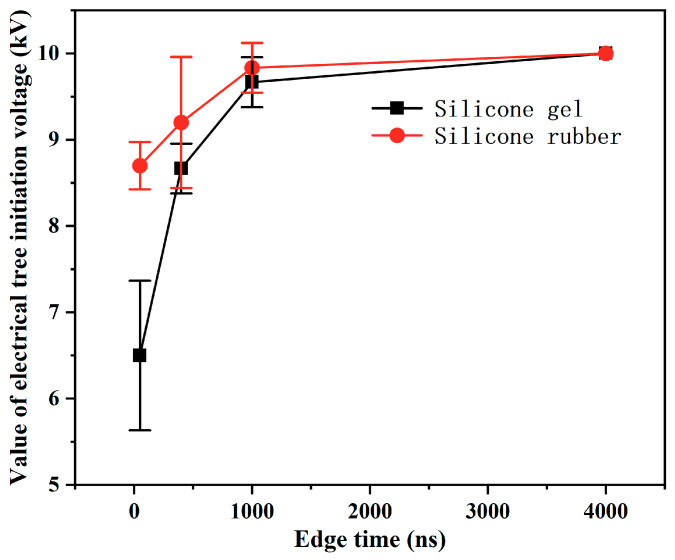
Relation between the initiation voltage of electrical trees and the edge time.

An amount of 1000 Hz and a 50% duty cycle negative square-wave pulse were applied to the silicone gel, and 5000 Hz and a 50% duty cycle negative pulse were applied to the silicone rubber. The pulse edge time of both was set to 50 ns, 400 ns, 1 μs, 4 μs, respectively, to explore the influence of the pulse edge time on the initiation and development of electrical trees. With the decrease in the edge time, the initiation voltage of electrical trees of silicone gel and silicone rubber decreased (Figure 10). Moreover, there is a turning point at around 1 μs of edge time. When the edge time is less than 1 μs, that is, when the edge time reaches the nanosecond level, the initiation voltage of electrical trees decreases rapidly in an exponential form as the edge time shortens. This demonstrates that nanosecond pulsed electric fields exert a significantly stronger impact on insulating materials compared to microsecond pulses.

**Figure 11:**
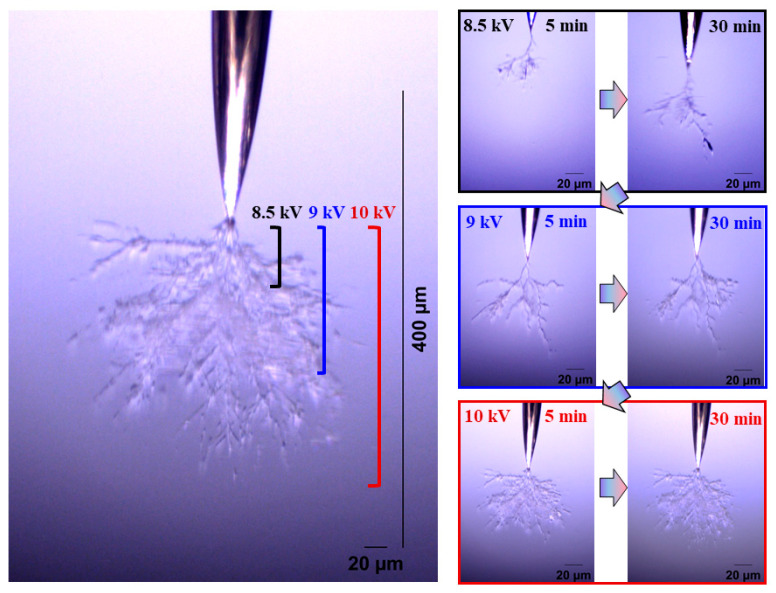
Electrical tree initiation and development process of silicone gel at edge time of 400 ns.

**Figure 12:**
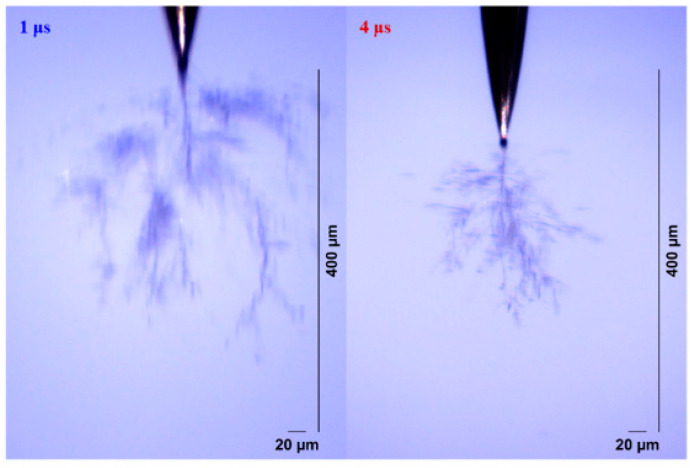
The morphology of the electrical trees of silicone gels at 10 kV and 30 min of applied voltage at edge times of 1 μs and 4 μs.

When the edge time is 400 ns, the electrical tree initiation and development of silicone gel are shown in Figure 11. Compared to the electrical tree in Figure 3 at 50 ns, the electrical tree at 400 ns is about 200 μm long when the voltage is increased to 10 kV for 30 min, which is about two-thirds of the length of the electrical tree at 50 ns. At 9 kV and 10 kV voltage levels, electrical trees at 400 ns are sparser and develop more slowly than those at 50 ns, with thinner branches. Due to the high electrical tree initiation voltage of 1 μs and 4 μs, which is as high as 9.5 kV, and the short development process, it is important to focus on the electrical tree morphology at the highest voltage level. Figure 12 shows the electrical tree morphology at 10 kV and 30 min of applied voltage for electrical trees with edge times of 1 μs and 4 μs. Comparing the electrical tree morphology at the same voltage level and time under 50 ns and 400 ns, it can be seen that the longer the edge time, the shorter the length of the electrical tree, the sparser the electrical tree, the fewer and thinner the branches, and the electrical tree morphology becomes dendritic.

**Figure 13:**
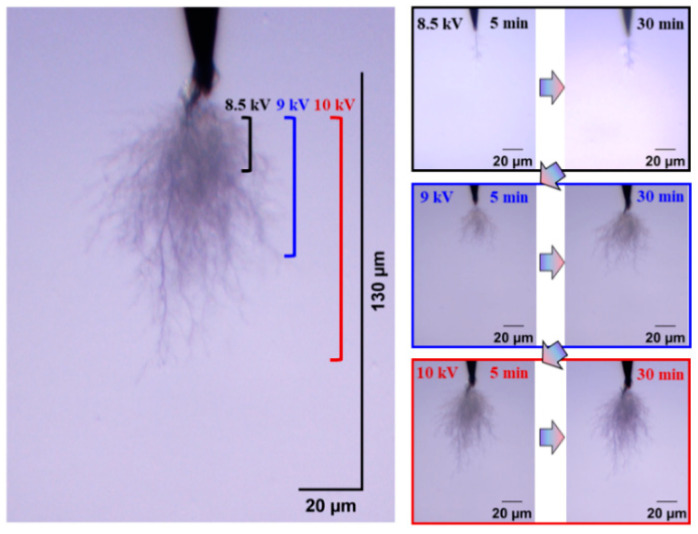
Electrical tree initiation and development process of silicone rubber at 400 ns.

**Figure 14:**
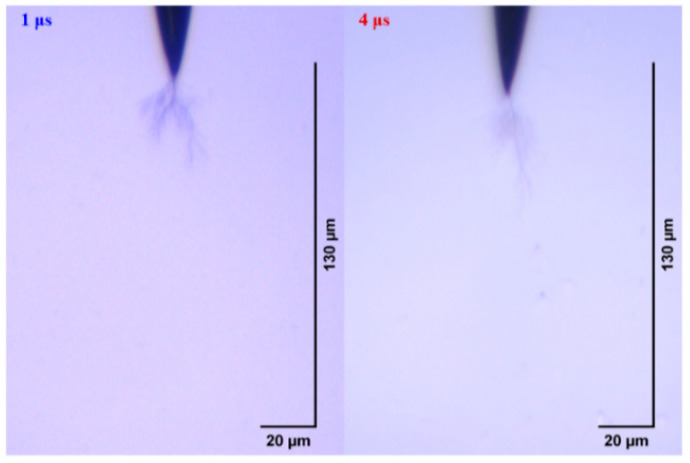
The morphology of the electrical trees of silicone rubbers at 10 kV for 30 min of applied voltage at edge times of 1 μs and 4 μs.

The electrical tree initiation and development process of silicone rubber at 400 ns is shown in Figure 13. Compared with the initiation and development of the electrical tree at 50 ns (Figure 5), the initiation of the electrical tree at 400 ns is a thin branch type. When the voltage is increased to 10 kV for 30 min, the length of the electrical tree at 400 ns is about two-thirds of that at 50 ns, and the electrical tree is sparser. Due to the high initiation voltage of the electrical tree at 1 μs and 4 μs, only the morphology of the electrical tree at the highest voltage level is of interest (Figure 14). The length and width of the electrical tree with an edge time of μs level are much smaller than those of ns level. The electrical tree under 1 μs is of the branch type, while the electrical tree under 4 μs is of the thin branch type.

The results of this section indicate that, for both silicone gel and silicone rubber, as the edge time decreases, the electrical tree initiation voltage becomes lower, the tree structures become denser, and the development speed is faster. Therefore, a reduction in the edge time promotes the growth of the electrical tree.

**Figure 15:**
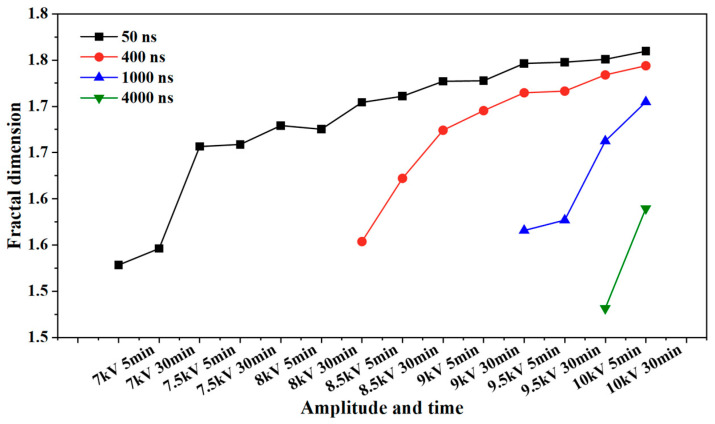
Variation in the electrical tree fractal dimension of silicone gel at different edge times with voltage amplitude and voltage application time.

**Figure 16:**
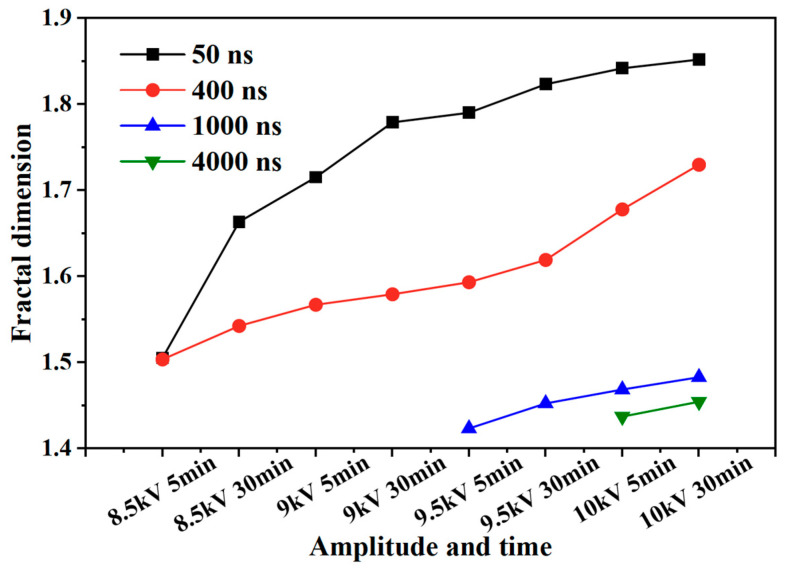
Variation in the electrical tree fractal dimension of silicone rubber at different edge times with voltage amplitude and voltage application time.

The development process of electrical trees at different edge times was quantified. Figure 15 and Figure 16 show the change in the fractal dimension of electrical trees of silicone gel and silicone rubber with the voltage amplitude and time under different edge times. As shown in the figure, when the pulse edge time decreases, the fractal dimension of electrical trees increases at the same voltage level and time, indicating that the growth density and complexity of electrical trees increase.

### 2.3. Effect of Temperature

**Figure 17:**
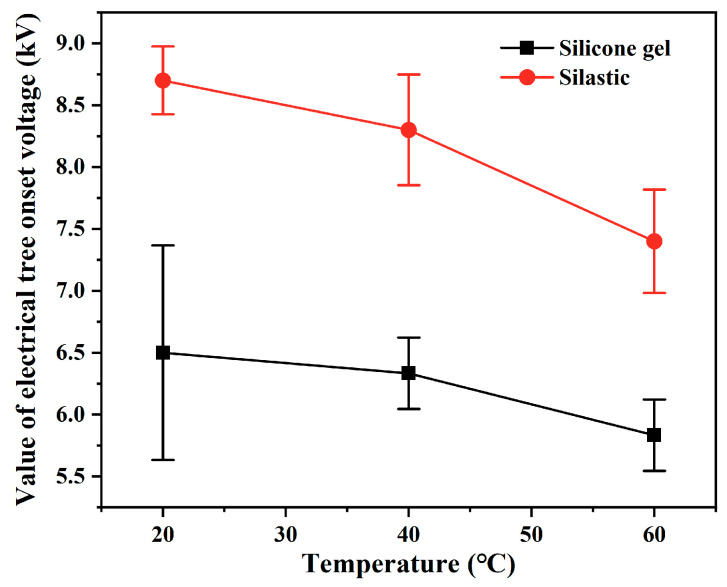
Relation between the mean value of the electrical tree onset voltage and the temperature.

High temperature is a critical operational condition for high-voltage semiconductor devices and a key factor influencing the insulation performance of their packaging [35,36,37,38]. Therefore, it is necessary to explore the impact of temperature on the electrical tree deterioration of silicone elastomers. For silicone gel, the parameters of the pulse voltage are a 1000 Hz repetition rate, 50 ns edge time, 50% duty cycle, and negative polarity. For silicone rubber, the parameters of the pulse voltage are a 5000 Hz repetition rate, 50 ns edge time, 50% duty cycle, and negative polarity. The temperature of the sample was maintained at 20 °C, 40 °C, and 60 °C to explore the effect of temperature on the initiation and development of electrical trees. Figure 17 shows the relation between the mean value of the electrical tree onset voltage and the temperature. It can be seen from the figure that when the temperature increases, the electrical tree initiation voltage of both silicone gel and silicone rubber decreases.

**Figure 18:**
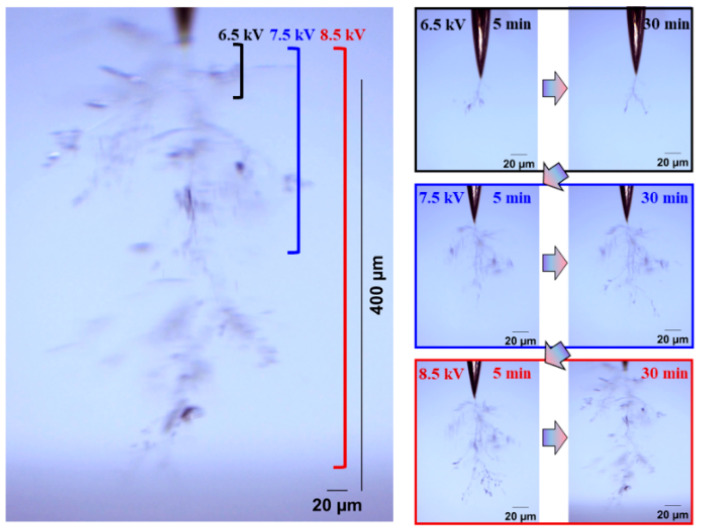
Electrical tree initiation and development process of silicone gel at 40 °C.

**Figure 19:**
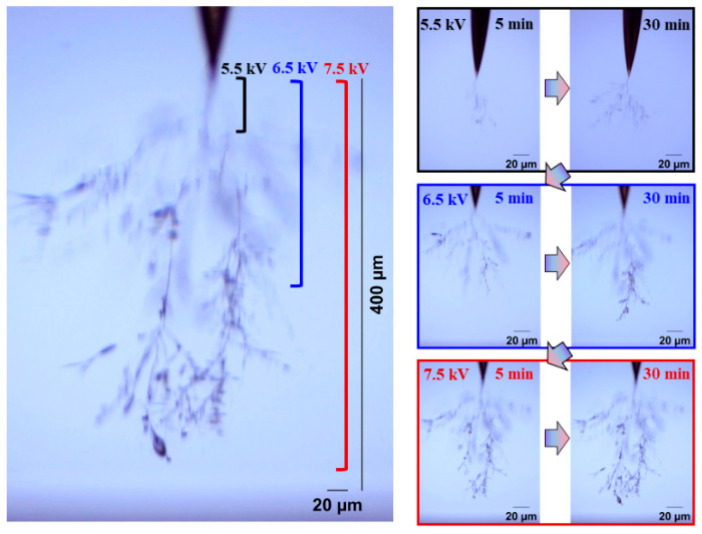
Electrical tree initiation and development process of silicone gel at 60 °C.

The development process and morphology of the electrical tree of silicone gel at 40 °C and 60 °C are shown in Figure 18 and Figure 19, respectively. Compared with the electrical tree at 20 °C in Figure 3, the electrical tree at higher temperatures has fewer branches and changes from a clumped shape to a dendritic shape. The electrical tree develops rapidly along the direction of the electric field, resulting in the highest breakdown voltage of 9 kV at 40 °C and 8 kV at 60 °C. At 60 °C, the electrical tree is denser and develops more rapidly than at 40 °C under the same voltage level.

**Figure 20:**
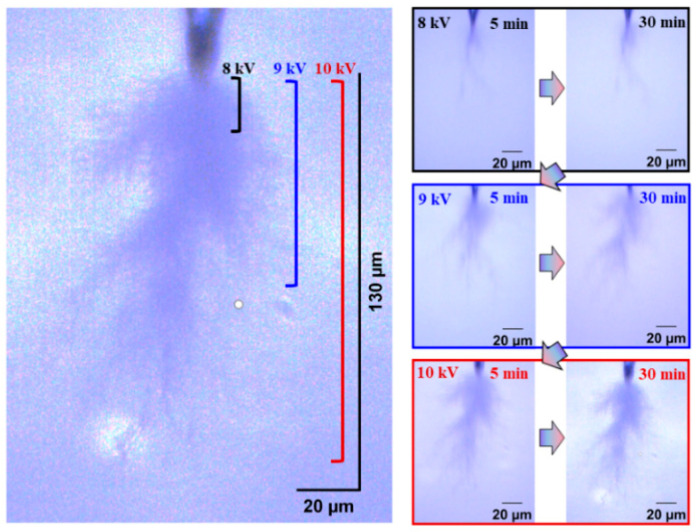
Electrical tree initiation and development process of silicone rubber at 40 °C.

**Figure 21:**
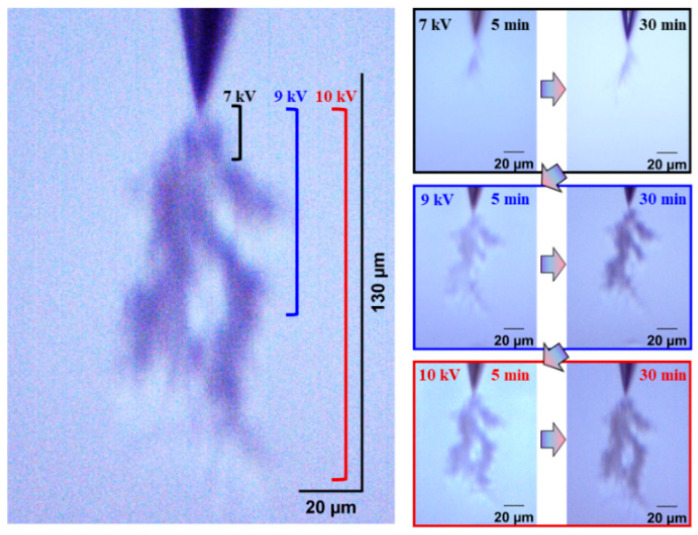
Electrical tree initiation and development process of silicone rubber at 60 °C.

Figure 20 and Figure 21, respectively, show the initiation and development of the electrical tree in silicone rubber at 40 °C and 60 °C. Compared to the electrical tree at 20 °C in Figure 5, the morphology of the electrical tree at higher temperatures is more ambiguous, meaning that there are more complex electrical trees overlapping together. The morphology of electrical trees at 40 °C was denser than that of the clumped shape, while at 60 °C, it exhibits a typical pine-like structure. The electrical tree grows more along the direction of the electric field, and the higher the temperature, the longer the electrical tree length.

**Figure 22:**
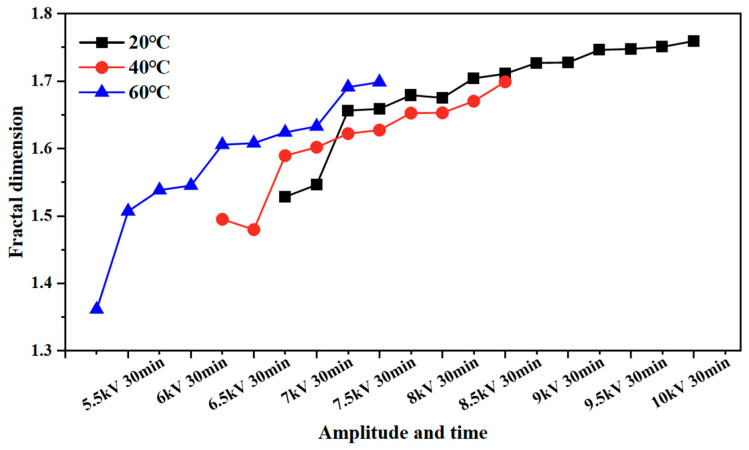
Variation in the electrical tree fractal dimension of silicone gel at different temperatures with voltage amplitude and voltage application time.

**Figure 23:**
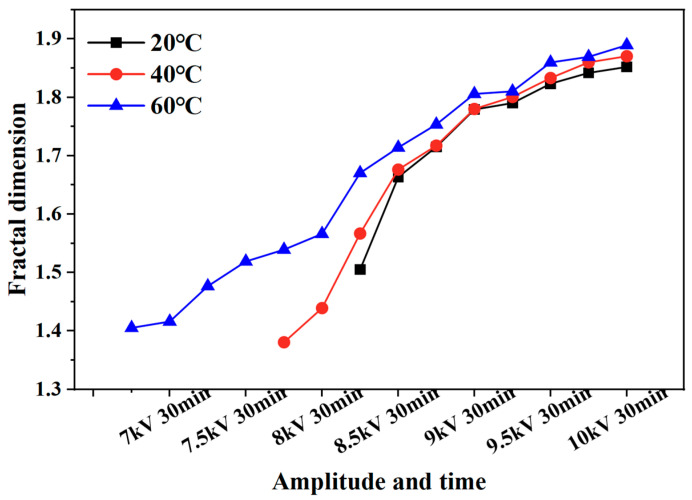
Variation in the electrical tree fractal dimension of silicone rubber at different temperatures with voltage amplitude and voltage application time.

Figure 22 and Figure 23 illustrate the variation in the fractal dimension of silicone gel and silicone rubber with respect to the voltage amplitude and time at different temperatures. For silicone gel, higher temperatures result in larger fractal dimensions before the voltage is increased to 7 kV for 30 min. However, after maintaining 7 kV for 30 min, the fractal dimension of the electrical tree at 40 °C becomes smaller than that at 20 °C. This phenomenon occurs because the rise in temperature causes the electrical tree structure to become more dendritic, leading to rapid growth toward the ground electrode along the electric field direction. The electrical tree has fewer lateral branches and is less complex. Compared to 40 °C, 60 °C not only promotes the rapid growth of electrical trees in the longitudinal direction, but also promotes the growth of lateral branches, resulting in an increase in the length and complexity of electrical trees and a higher fractal dimension. For silicone rubber, as the temperature increases, the electrical tree branches transform from a bushy shape to a denser pine-like shape, so the fractal dimension increases with the increasing temperature.

### 2.4. Mechanism of Deterioration Characteristics of Electric Branches

The increase in frequency, the decrease in edge time, and the increase in temperature all promote the initiation and development of electrical trees, so it is necessary to analyze the mechanism of each parameter separately.

#### 2.4.1. The Mechanism of Frequency

**Figure 24:**
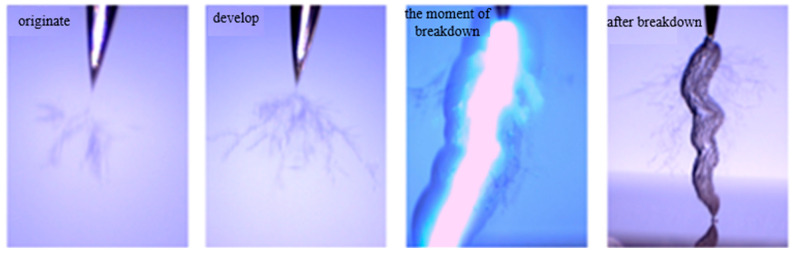
Electrical tree initiation and breakdown of silicone gel at frequency of 2000 Hz.

For silicone gel, when the frequency rises to 2000 Hz, the electrical tree of silicone gel begins to grow, the electrical tree in silicone gel grows rapidly, the phenomenon of bubble generation and recovery disappears, the electrical tree grows rapidly and penetrates the two poles, and intense discharge occurs at the moment of breakdown, forming a breakdown channel (Figure 24). The analysis of the causes suggests that due to the high frequency, the bubbles cannot recover in time before discharging again, resulting in the further development of the discharge channel on the original bubble channel, which leads to a sudden increase in the growth rate of electrical trees. When the frequency is greater than or equal to 3000 Hz, this process is more rapid and difficult to record. Therefore, the increase in frequency inhibits the self-recovery of electrical trees of silicone gel and promotes the growth of electrical trees. For silicone rubber, the increase in frequency and discharge frequency accelerates the growth rate of electrical trees.

#### 2.4.2. The Mechanism of Edge Time

**Figure 25:**
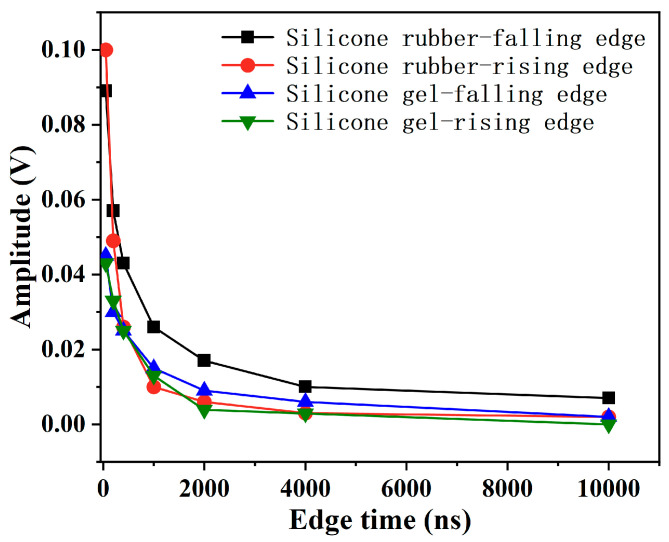
The effect of pulse edge time on the charge vibration of silicone gel and silicone rubber.

In the previous work of the research group, it was pointed out that the charge vibration at the pulse edge is one of the important factors causing insulation deterioration [39]. The charge vibration of silicone gel and silicone rubber at the pulse edge is also measured by the charge vibration platform under the pulse electric field, and the shorter the edge time, the stronger the charge vibration (Figure 25). Therefore, it can be inferred that when the edge time is shortened, the enhancement in charge vibration is an important reason for the decrease in the initiation voltage of electrical trees. In addition, according to the dielectric relaxation theory, due to the difference in mass and force between electrons and molecular segments, there is a huge difference in the response speed under extremely short pulses. This leads to the tendency of electrons to break away from their original position and undergo decoupling and dissociation, which enhances the activity of charge behavior and thus increases the rate of insulation deterioration.

#### 2.4.3. The Mechanism of Temperature

**Figure 26:**
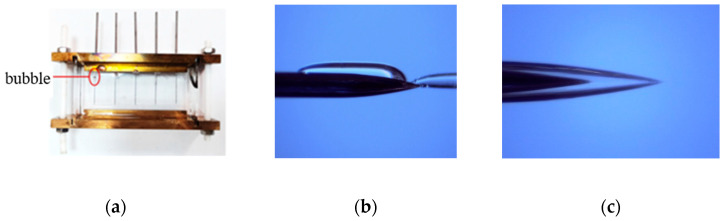
Air bubbles appear in silicone gel and silicone rubber when heated; (**a**) air bubbles appear in the silicone gel at a distance from the tip of the needle; (**b**) silicone gel bubbles move near the tip of the needle; (**c**) air gap appears at the silicone rubber tip.

When the temperature rises, both the silicone gel and silicone rubber samples show macroscopic changes. The silicone gel has bubbles visible to the naked eye at a distance from the needle tip (Figure 26a). And it gradually moves toward the needle tip with the increase in the voltage application time (Figure 26b). However, silicone rubber did not show visible bubbles at high temperatures, but there were clear air gaps at the tip of the needle under microscopic observation (Figure 26c). Due to the low dielectric strength of air, it is believed that the presence of air gaps is one of the important reasons for the decrease in the electrical tree initiation voltage of silicone elastomers at high temperatures.

## 3. Conclusions

This study set up an electric tree experimental platform under a pulsed electric field to test the electric tree characteristics of silicone gel and silicone rubber under different pulse frequencies, edge times, and temperatures; the development law of electrical trees under different working conditions is analyzed from three aspects: the average value of the initiation voltage of electrical trees, development structure, and fractal dimension.

The electrical tree initiation voltage of silicone gel is lower than that of silicone rubber under the same conditions, but silicone gel has a certain degree of self-recovery performance due to its solid–liquid two-state characteristics. With the increase in frequency, the electrical tree initiation voltage of silicone rubber and silicone gel decreased significantly, and the growth rate and damage area increased significantly. When the electric field frequency is higher than 2000 Hz, the electrical tree in the silicone gel develops rapidly and breaks down, while the electrical tree in the silicone rubber further develops under the promotion of the high-frequency electric field. After analysis, it is deduced that the frequency of partial discharge in the bubble increases with the increase in the frequency of the applied electric field, which promotes the development of electrical trees.

For silicone rubber and silicone gel, with the shortening of the edge time of the pulsed electric field, the initiation voltage of the electrical tree decreases significantly. The initiation voltage of the electrical tree under the nanosecond pulsed electric field is far less than that under the microsecond pulsed electric field and decreases exponentially. Moreover, the number of branches of electrical trees increases significantly with the decrease in the pulse edge time, which is mainly caused by being under a nanosecond pulsed electric field, while the reduction in the rise time significantly enhances the dielectric relaxation effect. Dielectric relaxation improves the local electric field concentration and energy transfer efficiency. The rapid changes in the nanosecond pulsed electric field accelerate the reorientation of dipoles, intensifying local polarization and energy dissipation.

The initiation voltage of electrical trees in silicone rubber and silicone gel decreases with the increase in temperature. For silicone rubber, higher temperatures increase the longitudinal length of electrical trees and reduce lateral branches. If the temperature is further increased, the lateral branches slightly increase. For silicone rubber, with the increase in temperature, the length of electrical trees in both the longitudinal and transverse directions increases, and the morphology changes from a sparse, clumped shape to a denser, pine-like shape. It was observed that an air gap appeared at the tip of the needle under high temperatures; it is speculated that the increased bubble discharge caused by high temperatures is one of the important factors promoting the generation and growth of electrical trees.

## 4. Materials and Methods

### 4.1. Preparation of Silicone Rubber and Silicone Gel

**Figure 27:**
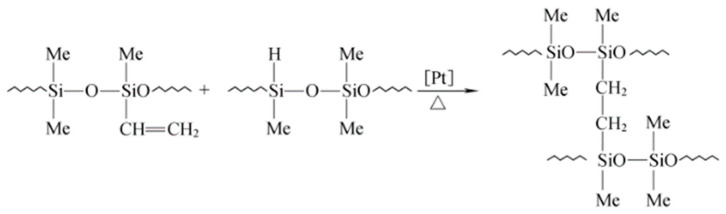
Principle of silicon–hydrogen addition reaction.

The silicone rubber and silicone gel used in the experiment are both dimethyl silicone, which are obtained through the two-component hydrosilylation reaction [40,41]; that is, crosslinking occurs through the addition of Si–H bonds in methyl hydrogenated silicone oil and vinyl groups in raw rubber catalyzed by chloroplatinic acid (Figure 27).

Mix the silicone gel and components A and B of silicone rubber at a mass ratio of 10:1, and the unvulcanized sample is liquid at this time. It is necessary to fully mix the silicone oil with various additives, and place the sample in a three-dimensional mixer for thorough spatial dynamic mixing. Put the mixed gel into the normal temperature vacuum box, vacuumize first, then the gas inside the sample will float on the liquid surface and then deflate to recover to atmospheric pressure. At this time, the bubbles on the liquid surface will break, and then vacuumize again and deflate. Repeat the operation for three cycles to preliminarily exhaust the gas inside the sample. Put the sample into the electric tree mold, put it into the 80 °C heating box, vulcanize the silicone gel for 30 min, and vulcanize the silicone rubber for 2 h.

### 4.2. Preparation of the Electrical Tree

**Figure 28:**
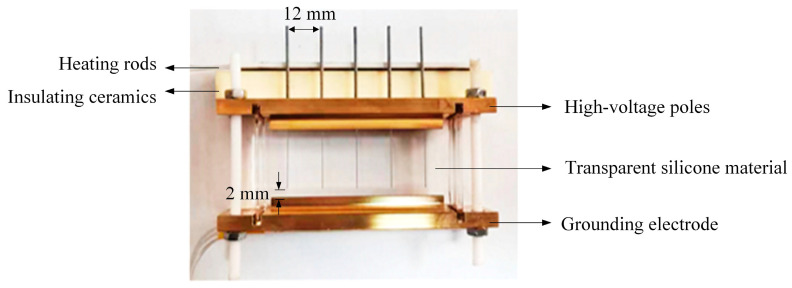
The specimen for silicone electrical tree experiment.

The needle electrode is the high-voltage electrode, and the brass plate in contact with the needle electrode is connected to the pulse power supply (Figure 28). The brass plate closer to the needle tip is grounded. The needle tip is 2 mm away from the ground plate, forming a non-uniform electric field with a needle–plate spacing of 2 mm. The radius of curvature of the needle tip is 0.2 μm, and the distance between the needle tips is 12 mm, allowing voltage to be applied to five needle–plate electrodes simultaneously to minimize errors.

Two brass plates can accommodate heating rods, which run through the width of the sample, ensuring uniform heating across the width of the sample and a consistent temperature across the five needle–plate electrodes. The temperature of the upper and lower plates can be adjusted separately to ensure that the temperature of the sample is uniform or adjust the temperature difference in the length direction.

### 4.3. Experimental Platform

**Figure 29:**
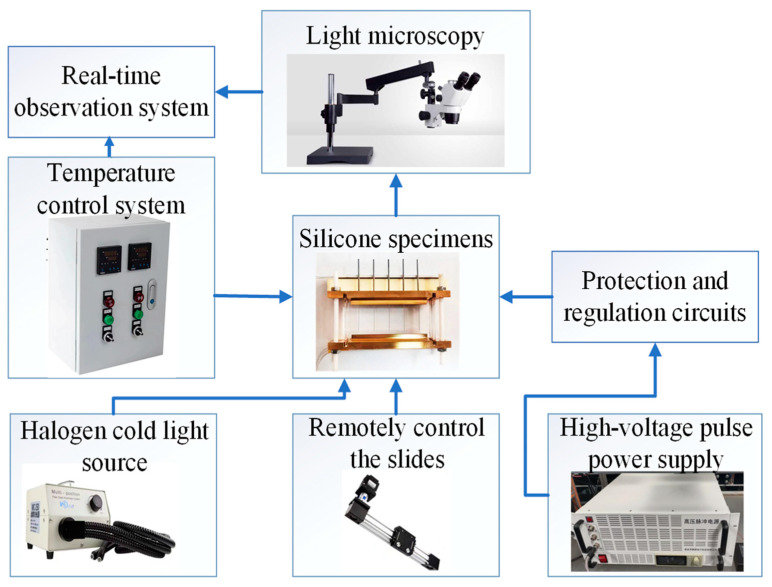
Schematic diagram of experimental platform for electrical tree under thermal coupled pulsed electric field.

The experimental setup comprises a high-voltage pulse power supply, protection and control circuits, silicone samples, a halogen cold light source, a light microscope, a remote-controlled slide rail, a temperature control system, and a real-time observation system (Figure 29).

The high-voltage pulse power supply outputs a continuous square-wave voltage with negative polarity, with a maximum output amplitude of 10 kV, a maximum output frequency of 10 kHz, an edge time of 50 ns, and a duty cycle of 50%. The protective resistor mainly has two functions: on the one hand, it can limit the current magnitude to protect the circuit when the sample breaks down; on the other hand, it can change the rise time of the applied high-voltage square-wave pulse. The correspondence between the resistance value and the edge time is shown in Table 1.

### 4.4. Experiment and Data Processing Methods

Due to the focus of this study on long-term operating conditions, the long-term accumulation method of increasing the voltage is more closely aligned with the actual operating conditions of semiconductor device packaging insulation. Specifically, the voltage is increased from 5 kV, and if no electrical tree is generated within 30 min, the voltage is increased by 0.5 kV. In this experiment, the initial length of the electrical tree is taken as 20 as a sign of the initial generation of the electrical tree. If the tree is generated within 30 min, record the voltage at this time as the initiation voltage of the electrical tree. Meanwhile, the voltage of the sample with electrical trees was continuously increased at the above rate to 10 kV, and the development image of the electrical tree was recorded every 5 min. The initiation and development of electrical trees at the five tips were observed simultaneously in each experiment to reduce experimental error.

The complexity of electrical tree development is quantified by fractal dimension. The electrical tree has a self-similar structure, and it is often difficult to distinguish the complexity of multiple electrical tree images by visual observation. However, fractal dimension can be quantitatively analyzed, and the larger the fractal dimension, the denser and more branches of the electrical tree image [42].

## 5. Patents

(1) Dongxin He, Cong Zhang. One-Step Solvent-Free Preparation of vinyl-terminated methylphenyl polysiloxane and Silicone Gel[P].Shandong Province:CN202411758089.8,2024-12-31.

(2) Dongxin He, Yuchao Li, Qingquan Li, et al. Phenyl Silicone Elastomer for High-Power Semiconductor Device Encapsulation and Preparation Method [P].:CN202410442814.4,2024-06-14.

## Figures and Tables

**Table 1:** Correspondence between resistance value and edge time.

**Resistance Value**	**Pulse Edge Time**
250 Ω	50 ns
1 kΩ	200 ns
2 kΩ	400 ns
5 kΩ	1 μs
10 kΩ	2 μs
20 kΩ	4 μs

## Data Availability

The original contributions presented in this study are included in the article. Further inquiries can be directed to the corresponding authors.
